# Nicotine Absorption Profile Among Regular Users of a Pod-Based Electronic Nicotine Delivery System

**DOI:** 10.1001/jamanetworkopen.2019.15494

**Published:** 2019-11-15

**Authors:** Jessica M. Yingst, Shari Hrabovsky, Andrea Hobkirk, Neil Trushin, John P. Richie, Jonathan Foulds

**Affiliations:** 1 Department of Public Health Sciences, Penn State College of Medicine, Hershey, Pennsylvania; 2 Penn State University College of Nursing, University Park, Pennsylvania; 3 Department of Psychiatry, Penn State College of Medicine, Hershey, Pennsylvania

## Abstract

This case series characterizes nicotine absorption among adults who regularly use a pod-based electronic nicotine delivery system.

## Introduction

JUUL is a popular electronic nicotine delivery system (ENDS)^1^ that contains a liquid with a high nicotine concentration (59 mg/mL). While there are concerns about the rapid increase in use of this potentially addictive product,^2^ there are no independent published data, to our knowledge, on its blood nicotine absorption profile. This case series aimed to characterize nicotine absorption among regular adult users of this product and to evaluate subjective effects associated with use.

## Methods

Current adult users of pod-based ENDS (n = 6), recruited via community flyers from November 2018 to May 2019, abstained from cigarette smoking for 4 days (carbon monoxide concentration verified as <8 ppm) and from any nicotine-containing product for at least 14 hours prior to attending a 1-day laboratory visit. Users completed baseline demographic and device questionnaires, including the Penn State Electronic Cigarette Dependence Index (PSECDI).^3^ Users were then instructed to puff on their own pod-based ENDS (nicotine concentration 59 mg/mL) every 20 seconds for 10 minutes. Blood was collected via catheter at baseline, while vaping (1, 2, 4, 6, 8, and 10 minutes), and after vaping (2 and 5 minutes after the last puff). Users rated withdrawal symptoms and subjective effects (scale of 0-100, with 100 indicating greatest effect) before and after vaping. Serum samples were analyzed for nicotine, cotinine, and 3-hydroxycotinine concentrations by liquid chromatography with tandem mass spectrometry.^4^ Outcomes included the maximal concentration, time to maximal concentration, and nicotine boost (maximal concentration minus the baseline nicotine level). Paired *t* tests were used to evaluate within-participants differences in subjective measures from before vaping to after vaping. Statistical significance was set at 2-sided *P* < .05. Statistical analysis was performed using SAS version 9.4 (SAS Institute Inc).

This study was approved by the Penn State College of Medicine institutional review board, and all participants provided written informed consent. Data are reported using the reporting guideline for case series.

## Results

Participants were 83.3% white, 33.3% male, and had a mean (SD) age of 37.8 (15.8) years (Table). The mean (SD) PSECDI score was 14 (3.7). The mean (SD) maximal concentration of nicotine was 31.1 (13.2) ng/mL, the mean (SD) time to maximal concentration was 8.7 (1.6) minutes, and the mean (SD) nicotine boost obtained was 28.6 (9.8) ng/mL. The mean (SD) nicotine boost at 4 minutes was 12.9 (9.8) ng/mL. The nicotine absorption profile for each participant is displayed in the Figure. After use, participants reported lower anxiety (mean [SD] score, 52.7 [38.4] before use to 7.5 [14.6] after use; *t* = 2.76; *P* = .04) and lower craving (mean [SD] score, 74.7 [36.7] before use to 11.2 [19.6] after use; *t* = 3.53; *P* = .02).

**Table.** Participant and Device Characteristics

Characteristic	Participant No.						Overall
	1	2	3	4	5	6	
Age, y^a^	48-57	18-27	18-27	48-57	18-27	48-57	Mean (SD), 37.8 (15.8)
Current smoker	No	No	No	Yes	Yes	No	33.3% Yes
Times/d^b^	120	50	30	15	5	10	Mean (SD), 34.2 (43.4); median (IQR), 22.5 (8.75-67.5)
PSECDI score	17	17	17	13	12	8	Mean (SD), 14 (3.7)
Flavor used during study	Mango	Mango	Mango	Strawberry lemonade^c^	Mango	Menthol	66.6% Mango
Nicotine concentration used during study, mg/mL^d^	59	59	59	59	59	59	Mean, 59
Nicotine metabolite ratio^e^	0.89	0.38	0.34	0.93	0.011	0.47	Mean (SD), 0.50 (0.35)
Nicotine boost obtained, ng/mL	38.8	28.2	22.6	16.3	41.7	23.8	Mean (SD), 28.6 (9.9)

Abbreviations: IQR, interquartile range; PSECDI, Penn State Electronic Cigarette Dependence Index.

^a^ Ages are shown in 10-year categories for participant confidentiality.

^b^ Each “time” is defined as use lasting approximately 15 puffs or approximately 10 minutes.

^c^ Participant used a non-JUUL-brand pod (Ziip Pods).

^d^ As indicated by the product manufacturer (not measured).

^e^ Calculated by dividing the baseline 3-hydroxycotinine level by the baseline cotinine level.

**Figure.**
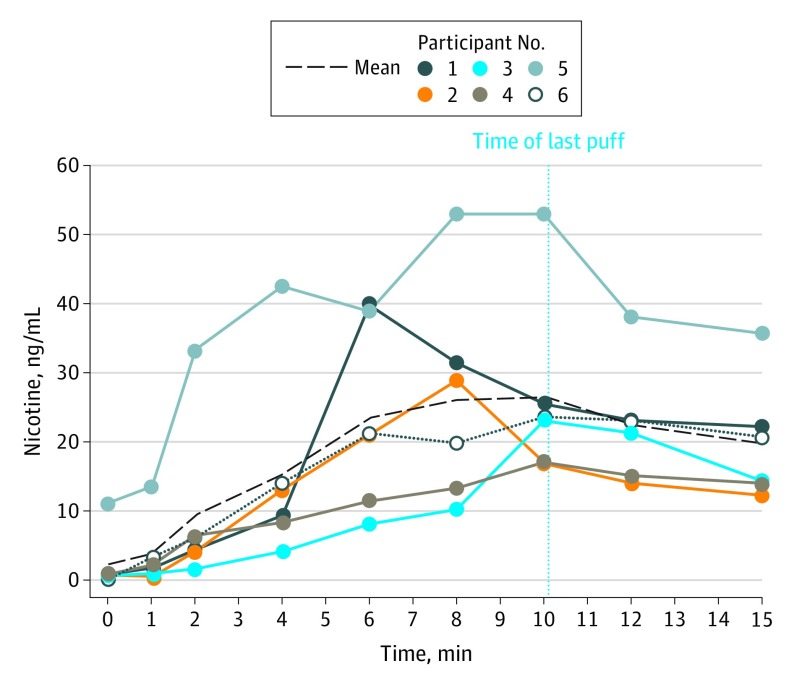
Serum Nicotine Levels for Individual Participants, Collected During Active Vaping

One participant (participant 4) who used a non-JUUL pod obtained the lowest nicotine boost (16.3 ng/mL). Additionally, participant 5 had a greater baseline nicotine level and obtained a greater nicotine boost compared with other participants. This could be due to the participant’s low nicotine metabolite ratio (0.011 [calculated by the dividing the baseline 3-hydroxycotinine level by the baseline cotinine level]), suggesting that this participant metabolizes nicotine at a very slow rate.

## Discussion

Among experienced users who took 30 puffs in 10 minutes, the pod-based ENDS delivered a mean nicotine boost of 28.6 ng/mL in a mean of 8.7 minutes. This is higher and faster than the mean nicotine boost obtained from “cigalike” ENDS devices (puff activated and similar size and shape as a traditional tobacco cigarette; 1.8 ng/mL, 10 minutes) and advanced ENDS devices (button-activated with larger battery; 10.8 ng/mL, 12.1 minutes) using the same puffing schedule.^4^ These users also self-reported higher nicotine dependence on the PSECDI, compared with 3609 experienced long-term users of other ENDS devices (mean PSECDI score, 8.1).^3^

This study is the first, to our knowledge, to show that JUUL delivers a higher and faster boost in blood nicotine than has been reported for most other ENDS devices.^4,5,6^ Limitations are the small sample size and that ENDS users may not typically take 30 puffs in 10 minutes. However, its use produced a mean (SD) nicotine boost of 12.9 (9.8) ng/mL after only 12 puffs in 4 minutes, a rate suggestive of pulmonary absorption. Compared with studies reporting the nicotine boost obtained after smoking 1 cigarette, this product’s nicotine delivery was similar.^4,6^ The nicotine delivery capabilities of this ENDS device may contribute to its addictiveness as well as its ability to compete with cigarettes for market share.
